# Bond Strength of Reline Materials to 3D-Printed Provisional Crown Resins

**DOI:** 10.3390/polym15183745

**Published:** 2023-09-13

**Authors:** Jorge Palavicini, Sherrod L. Quin, Wael Zakkour, Karim Zakkour, Safa Manafi Varkiani, Xiaoming Xu, Nathaniel C. Lawson, Amir Hossein Nejat

**Affiliations:** 1 Department of Prosthodontics, Louisiana State University Health Science Center, School of Dentistry, New Orleans, LA 70119, USA; 2 Department of Comprehensive Dentistry, Louisiana State University Health Science Center, School of Dentistry, New Orleans, LA 70119, USA; 3 Private Practice, Lynchburg, VA 24501, USA; 4 Department of General Surgery, Saint George University of Beirut, Beirut 1100-2807, Lebanon; 5 Henry M. Goldman School of Dental Medicine, Boston University, Boston, MA 02118, USA; 6 Department of Oral and Craniofacial Biology, Louisiana State University Health Science Center, School of Dentistry, New Orleans, LA 70119, USA; 7 Division of Dental Materials, University of Alabama at Birmingham School of Dentistry, Birmingham, AL 35233, USA

**Keywords:** additive manufacturing, CAD/CAM technology, dental materials, dental crown, provisional crown, bond strength

## Abstract

(1) Purpose: The aim of the present study was to compare the bond strength between two 3D-printed resins designed for long-term provisional crowns and three different reline materials. (2) Materials and Methods: Rectangular specimens were prepared from two 3D-printed resins (Envision Tech and NextDent C&B) and a conventional self-cure PMMA. Transparent tubes filled with three different reline materials including composite resin, Bis-acryl, and PMMA were bonded to the 3D-printed specimens (n = 11 per group, total of 6 study groups). Tubes filled with PMMA were bonded to the prepared PMMA specimens which served as the control group (n = 11, control group). The specimens were subjected to a shear bond strength (SBS) test, and mode of failure was recorded using light microscopy. Statistical analysis was performed using a one-way ANOVA and post hoc Tukey’s tests (alpha = 0.05). (3) Results: The highest SBS value was achieved to both 3D-printed materials with the PMMA reline material. The bond to both 3D-printed materials was lower with Bis-acrylic or composite resin relines in comparison to that with PMMA (*p*-value < 0.05). No significant difference was found between the control PMMA group and either 3D-printed material when relined with PMMA (*p*-value > 0.05). (4) Conclusion: The tested 3D-printed resins achieved a clinically acceptable bond strength when relined with PMMA.

## 1. Introduction

Introduction of the computer-aided design/computer-aided manufacturing (CAD/CAM) technology has revolutionized the manufacturing methods in the field of dentistry and has allowed for the development of a wide range of dental materials to be used as either a temporary or final prosthesis. Traditionally, CAM technologies were subtractive and milled the prosthesis from a pre-made blank. However, the introduction of additive manufacturing (AM), commonly referred to as 3D-printing technology, into the field of dentistry has modified the conventional techniques used in fabrication of dental prostheses. Commonly used 3D-printing technologies in dentistry are stereolithography (SLA) and digital light processing (DLP), both of which utilize a vat filled with photopolymerizable resin. The resin is cured layer by layer by the light source, and then the printed structure undergoes a series of post-processing steps in order to become fully cured [1]. Various parameters of 3D printing can affect the mechanical and fatigue properties of the printed parts according to the manufacturing technique used [2]. When compared with subtractive technology, 3D printing has less waste of material (not considering the material used for post-cure processing) and fewer restrictions considering the geometry of the manufactured object [1]. The polymers used to fabricate 3D-printed dental restorations are composed of dimethacrylate systems containing methyl methacrylate, bisphenol A-glycidyl dimethacrylate (Bis-GMA), triethylene glycol dimethacrylate (TEGDMA), and urethane dimethacrylate (UDMA) [3].

Provisional crowns (also called interim or temporary crowns) are commonly used in dental practices to serve the patient between the tooth preparation and the delivery of the definitive prosthesis in order to prevent tooth migration, further tooth loss due to function, food impaction, tooth sensitivity, gingival overgrowth, and provide acceptable esthetics. In addition to tooth-based restorations, an implant-supported prosthesis would require an appropriate provisional restoration; fabrication of an appropriate interim implant-supported prosthesis would be crucial for a proper gingival tissue contour. Since provisional restorations would be subjected to chewing forces and would be visible, an appropriate and clinically acceptable provisional restoration needs to meet the criteria for mechanical and esthetic properties. These properties would be more critical in patients with parafunctional habits, long-span fixed prostheses, full mouth oral rehabilitations in need of changes in the vertical dimension of occlusion, and patients in need of temporomandibular joint dysfunction therapies. Moreover, due to the fact that provisional restorations may be in contact with gingival and periodontal tissues, it is essential that these materials be biocompatible and provide an acceptable tissue response [4,5]. 

In clinical situations such as full-mouth rehabilitations and full-arch implant-supported immediate loading, provisional restorations may stay in function for several months prior to replacement with the definitive prostheses. Hence, provisional materials are needed to maintain their properties over time in the oral cavity. It should also be noted that in these long-term provisional restorations, the restoration needs to be capable of being repaired on the external surface or be relined on the intaglio surface [5]. Both relining and repair require a sufficient bond to the provisional material. 

Various materials are being used to fabricate provisional restorations. In general, the provisional materials can be classified into two broad categories of resin-based composites and polymer-based materials based on their chemical composition [6,7]. An example of a resin-based composites is bis-acrylic, which would be the material of choice for single-unit provisional restorations. Bis-acrylic materials contain cross-linking monomers, such as Bis-GMA, TEGDMA, and UDMA, as well as silica fillers. The advantages of the bis-acrylic provisional material include the ease of use due to the paste–paste system of delivery, low exothermic reaction, which has a lower chance of causing soft tissue and pulpal tissue irritation and damage, and low shrinkage of the material during setting, which enhances the fit of the provisional restoration. However, this material is not appropriate for multiple unit restorations as it lacks the required mechanical properties. Also, bis-acrylic restorations do not function well over longer periods of time, which makes them inappropriate for long-term provisional restorations. In terms of color stability, bis-acrylic provisional material is less color-stable than polymer-based restorations [7]. Bis-acrylic provisional has low bond strength to the same material, which makes the repair process difficult and challenging. Additionally, the thick oxygen-inhibited layer that occurs upon setting makes the surface hardness of these materials inferior. Furthermore, repairing these materials is challenging. Hence, bis-acrylic restorations are more suitable for short-term single-unit restorations. In terms of cost, resin-based provisional materials are generally costlier than polymer-based ones [6]. 

The most commonly used polymer-based provisional material is polymethylmethacrylate (PMMA), which is the preferred material for long-span and long-term provisional restorations due to its mechanical properties, ease of repair, color stability, and low cost [6]. However, the polymerization reaction of PMMA is exothermic and has the potential to irritate the pulp and gingival tissue and cause volumetric shrinkage, which would cause dimensional changes and may affect the fit of the restoration. In addition, the odor is not pleasant for many patients [8,9,10,11]. PMMA is a long-chain linear polymer with minimal crosslinking [3]. Another type of polymer-based provisional material is polyethyl methacrylate (PEM); when compared with PMMA, PEM has higher biocompatibility, produces less heat during the setting reaction, and undergoes less volumetric shrinkage during setting. However, PEM is less color-stable and has mechanical properties lower than those of PMMA. As a result, PEM is more suitable as a reline material than PMMA is. When using PMMA as the shell and relining it with PEM, high bond strength between the shell and reline material is expected [6].

The introduction of 3D-printing technology has had a significant impact on the field of restorative dentistry [12]. Three-dimensional printing offers a relatively fast and inexpensive method to fabricate dental models, custom trays, surgical guides, partial or complete dentures, splints, and provisional and final restorations [13]. Moreover, these resins have been innovatively modified by adding bioactive glasses to provide bioactive properties [14]. The provisional restorations can be designed through digital planning, allowing the contours of the provisional restorations to best provide an esthetic appearance, test an occlusal design, and shape soft tissue. The mechanical properties of several 3D-printed resins appear promising for the use of long-term provisional restorations. Tahayeri et al. [15] investigated the mechanical properties and degree of conversion of printed resins in comparison to Jet acrylic and Integrity; they concluded that mechanical properties of 3D-printed provisional resins are sufficient for intraoral use. Park et al. [16] found that 3D-printed resin has similar wear resistance when compared with self-cure and milled PMMA. Since 3D-printed resins are available in limited shades, there might be a need to individually characterize the long-term provisional restorations. While the mechanical properties of 3D-printed resins are found to be clinically acceptable, the ease of repair and relining of these materials need to be tested as an important feature of long-term provisional material [17]. The chemical reaction between the 3D-printed provisional crown and the reline material occurs through the bond between unreacted methyl methacrylate, Bis-GMA, TEGDMA, and UDMA monomers of the 3D-printed provisional material and the methacrylate monomers of PMMA or Bis-GMA, TEGDMA, and UDMA monomers in bis-acrylic [3]; however, the bond strength between various monomers and 3D-printed provisional material need to be tested. Holmer. et al. [18] found that shear bond strength of resin cements is significantly higher than that of a resin modified glass ionomer to 3D-printed temporary resin specimens. Albahri et al. [19] evaluated the shear bond strength of various repair materials to a 3D-printed stereolithography resin and found no significant difference between the tested repair materials. Lankes et al. [20] tested the effect of different cleaning methods and air-abrasion parameters on the shear and tensile bond strength of 3D-printed temporary resin to resin cement. They reported the highest bond strength when the surface was treated with alumina particles at 0.4 MPa. Song et al. [21] has shown the dynamics of cement setting through THz spectroscopy and found that cross-linking of cement starts 3 h after mixing, during which the gelation of the cement is dominant and then followed by interfacial growth between the polymers in cement and the glass composite. They found that the bulk of the cementation reaction completes in 24 h [21]. 

The aim of the present study was to measure and compare the shear bond strength of two commercially available 3D-printing resins (EnvisionTec and NextDent) approved for long-term provisionals. The primary hypothesis was that the bond strength between the 3D-printed resins and PMMA reline material would be significantly higher than the shear bond strength to bis-acryl and composite reline materials. The secondary hypothesis was that the bond strength to either type of 3D-printed resin would be similar regardless of the type of reline material. 

## 2. Materials and Methods

### 2.1. Specimen Preparation

In this study, specimens were made from two commercially available 3D-printed resins designed for long-term provisionals, including E-Dent 400 C&B MFH (Envision-TEC, Dearborn, MI, USA) and NextDent C&B MFH (Vertex-Dental B.V., Soesterberg, The Netherlands). Both 3D resins are classified as class IIa by the FDA [6]. The control group was self-cure PMMA. A list of the materials used in the present study is provided in Table 1.

Specimen geometry in the form of a square was digitally designed in CAD software (Meshmixer software, Version 3.5, Autodesk Inc., San Francisco, CA, USA) and then exported as a Standard Tessellation Language (STL) file format. The STL files were then placed in a 3D printer’s CAM software (Envision One RP 1.20.4470). The objects were placed in horizontal orientation, and supports were created to be able to print the samples. For each type of 3D-printed resin, 33 squares of 12 × 12 mm in dimensions and thickness of 2 mm were printed in a D4K Pro Dental 3D printer (Envision-TEC, Dearborn, MI, USA). Post-processing followed the manufacturer’s recommendation and included rinsing the 3D-printed samples with isopropanol in an ultrasonic bath and completing the polymerization using a light polymerization chamber (PCA 100, Envision-TEC). In addition, eleven extra squares were printed and embedded inside poly-vinyl siloxane in order to create a matrix for fabrication of samples from self-cure PMMA (Alike, GC, Alsip, IL, USA) with similar dimensions. For these samples, the liquid and powder were mixed according to the manufacturer’s recommended proportions and were poured into the polyvinyl siloxane matrix and allowed to self-cure according to the manufacturer’s recommended setting time. Hence, a total of 77 base squares were fabricated for this study. Following the fabrication of the samples, each sample underwent a series of polishing in order to standardize the surface roughness, which included 60 grit SiC paper as the first step, followed by 120 grit paper, 320 grit paper, 600 grit paper, and finally 1200 grit SiC paper. The SiC papers were all mounted on a grinding machine (LaboPol-5, Struers, Cleveland, OH, USA), and the polishing was performed using a speed of 200 rpm for 15 s and under running water to remove the debris and keep the papers clean. After polishing, each sample was placed in deionized water and placed in an ultrasonic bath in order to remove the remnants of the silicon carbide sandpapers. The samples were then left to dry at room temperature prior to the bonding procedure. 

### 2.2. Study Groups

Each 3D-printed resin was divided into three groups according to the type of reline material, resulting in six groups in addition to the control group (n = 11 per group): Group EP used the E-Dent resin specimen as the base and was relined with PMMA (Alike); Group EB used the E-Dent resin specimen as the base and was relined with bis-acrylic (Integrity Multicore, Dentsply Sirona, Charlotte, NC, USA); Group EC used the E-Dent resin specimen as the base and was relined with composite resin (Filtek Supreme, 3M ESPE, Saint Paul, MN, USA); Group NP used the NexDent resin specimen as the base and was relined with PMMA; Group NB used the NextDent resin specimen as the base and was relined with bis-acrylic; Group NC used the NextDent resin specimen as the base and was relined with composite resin; Group PP served as the control group and used the PMMA specimen as the base and was relined with PMMA. Prior to placement of reline material on the base, in groups EP and NP, the surface was wetted with a PMMA monomer (Alike). In EC and NC groups, ScotchBond Universal adhesive (3M ESPE) was applied to the surface for 20 s and air thinned. 

### 2.3. Bonding Procedure

In order to place a cylinder of the reline material on top of each specimen base, a transparent tygon tube with an internal diameter of 2 mm and height of 3 mm was used. While placed on the base specimen, each tube was filled with the intended reline material (in groups EP, NP, and PP, the tube was filled with PMMA, in groups EB and NB, the tube was filled with bisacrylic, and in groups EC and NC, the tube was filled with composite resin). The reline material was light-cured in groups EC and NC for 40 s with a dental light-cure unit (Bluephase 20i, 1200 mW/cm^2^, Ivoclar Vivadent, Schaan, Liechtenstein). The specimens were then allowed to complete the polymerization process for 24 h in the incubator at 37 °C (24). The tygon tubes were left on the base and were removed right before the shear bond strength test. 

### 2.4. Shear Bond Strength (SBS)

The specimens were attached to a shear-testing jig designed for a universal testing machine and then subjected to shear forces (universal testing machine, 5566, Instron, Norwood, MA, USA) using a flat blade with crosshead speed of 1 mm/min until failure. The bond strength values (MPa) were calculated by dividing the load (N) at failure by the cylinder’s surface area (mm^2^). Then, the specimens were examined under an optical microscope (Eclipse 50i, Nikon, Tokyo, Japan) to determine the mode of failure. The mode of failure was categorized into adhesive, cohesive (either in the base or reline material), and mixed. 

### 2.5. SEM Evaluation

A representative specimen from each type of failure mode was selected to obtain a scanning electron microscopy (SEM) image. The selected specimens were mounted on a holder and coated using an ion sputter coater. Then, the selected samples were placed in a Hitachi S-2700 scanning electron microscopy machine (Hitachi, Tokyo, Japan), and high-resolution images were obtained under 100× magnification.

### 2.6. Statistical Analysis

Shear bond strength (MPa) was calculated for each group and is presented as the mean and standard deviation. The data were analyzed using one-way ANOVA and post hoc Tukey’s tests with the significance level set at 0.05 and using SPSS version 26.0 software (IBM SPSS Statistics, Armonk, NY, USA).

## 3. Results

### 3.1. Shear Bond Strength Results

**Figure 1:**
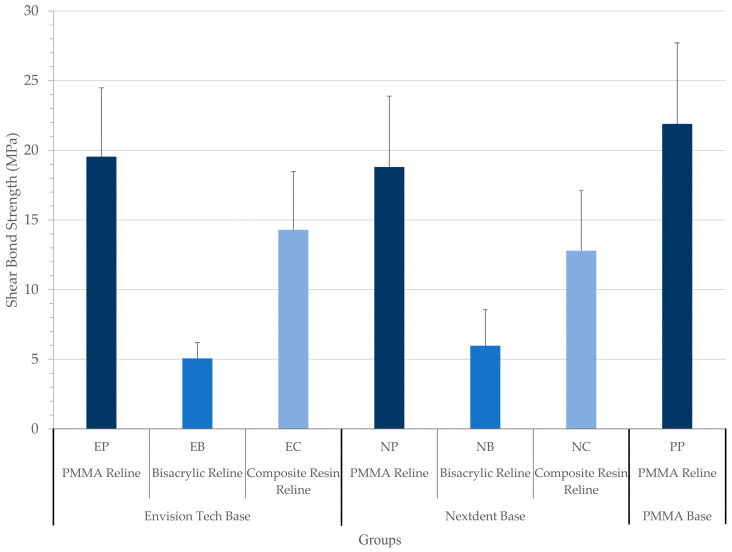
Shear bond strength of the studied groups.

The shear bond strength (SBS) values of the tested samples are summarized in Table 2 and demonstrated in Figure 1. The highest SBS value was found in the control group (PP) with a mean value of 21.89 MPa, followed by those of group EP with a mean value of 19.54 MPa, group NP with a mean value of 18.80, group EC with a mean value of 14.29, group NC with a mean value of 12.79, group NB with a mean value of 5.97, and group EB with a mean value of 5.06. According to the statistical analysis, the SBS of the three groups of each base and the control group were significantly different (*p* < 0.001). Post hoc tests revealed that the differences between the PP and EB, EC, NB, or NC were statistically significant, and no significant difference was found between EP and PP (*p* = 0.597) or between NP and PP (*p* = 0.406). Since the highest SBS was obtained using PMMA as the reline material in all groups, the SBSs of PMMA reline material to different base materials including E-Dent (EP), NextDent (NP), and PMMA (PP) were compared separately, and no significant differences were found (*p* > 0.05) 

### 3.2. Mode of Failure Results

**Figure 2:**
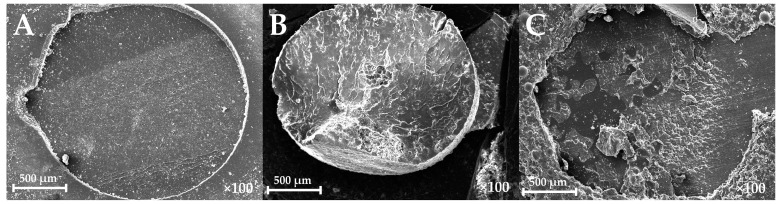
(**A**) Adhesive failure mode: no remnants of the reline material are left on the surface of the specimen; (**B**) cohesive failure mode: the reline cylinder is broken within the cylinder and left on the whole area of the bonding surface; (**C**) mixed failure mode: some remnants of the reline material are left on the bonded surface, covering a part of the surface, and the rest of the bonded surface has the base material exposed.

The mode of failure of the tested groups is summarized in Table 3. Adhesive was the dominant mode of failure in group EB (91%), group NB (82%), and group NC (55%). In group EP (73%), group NP (73%), group PP (91%), the most common mode of failure was adhesive. In other words, the most common mode of failure in the groups relined with PMMA was mixed, while with the other reline materials, the dominant mode of failure was adhesive (Table 3). Sample SEM pictures are shown in Figure 2.

## 4. Discussion

In this study, shear bond strength between two FDA-cleared 3D-printed provisional resins and three different reline materials was tested. The first hypothesis was that the highest bond strength would be found in 3D-printed resins with PMMA as the reline material when compared with bis-acryl and composite reline materials. The second hypothesis was that there will be no differences between the two commercial 3D resins regardless of the type of reline materials. Considering the results of the present study, both hypotheses were accepted, as the highest shear bond strength was observed when the 3D-printed temporary resin was attached to the self-cure PMMA; in addition, both tested 3D-printed resins revealed similar bond strength values when attached to the self-cure PMMA. 

Since the chemical composition of the 3D-printed resins is not fully clear and there is no clear guideline on how to reline the provisional crowns fabricated with these resins, in the present study, various materials were used to test the SBS to two types of 3D-printed resins. It was found that PMMA resulted in the highest SBS value regardless of the type of base. 

Chen et al. [22] found that the highest shear bond strength was obtained when the provisional material was repaired with repair material with a similar chemical skeleton. In the present study, it was found that the SBS bond between the 3D-printed resins and self-cure PMMA was higher than that with other reline materials and was comparable to the bond observed when the base was also PMMA; this may suggest similarity between the chemical composition of both 3D-printed resins and self-cure PMMA. According to the data safety sheet, NexDent C&B consists of methacrylate oligomers and glycol methacrylate; Tahayeri et al. [15] also mentioned that the NexDent C&B resin is at least 90% methacrylic oligomers with no fillers. In addition, Jeon and Kim [23] mentioned that Nexdent C&B is a monomethacrylate-type material that is similar to Alike and is compatible with the present findings. However, they found that SBS between 3D-printed resins and a bis-acryl reline was higher than the bond with a self-cure PMMA reline material. This finding is in contrast with that in the present study, where the lowest SBS was obtained with a bis-acryl-type material. Jeon and Kim [23] mentioned that a bis-acryl reline material creates a complex network that improves the bond in comparison to that with the PMMA reline material. On the other hand, Chen et al. [22] reported the lowest SBS when a PMMA base was relined with bis-acryl-type materials, which would be compatible with the present findings assuming a chemical similarity between the tested 3D-printed resins and PMMA.

It has been shown that surface treatment has the potential to improve the bond strength to provisional materials, although it will add steps, chairside time, and possibly cost to the reline process. Goncalves et al. [17] showed that composite resin can be used as reline material for bis-acryl and PMMA when a composite primer is applied to the surface. Alternatively, a combination of a methyl methacrylate monomer and bonding agent could be used instead. Determining the most practical reline material with the fewest steps and lowest cost to obtain a reliable bond to the provisional restoration is crucial. In the present study, the surface of the PMMA groups was refreshed with a monomer before applying a self-cure PMMA; this could be a contributing factor in observing higher shear bond strength in these groups as the monomer caused a partial dissolution of the surface material and possibly better and deeper connection between the two materials [24]. 

Fabrication of provisional shells prior to tooth preparation has many advantages, including reduced chairside time, digitally assisted design, less unfavorable odor caused by PMMA, and possibly a reduction in trauma to the tissue from the exothermic reaction (due to the decreased amount of time needed to reline in comparison to that when fabricating the entire provisional in the mouth) [25,26]. In addition, prefabricated provisional restorations, regardless of the fabrication method (milled, heat cured, or 3D-printed), may improve the mechanical properties of the provisional restoration by increasing the monomer conversion percentage [15]. Selection of the reline material for relining provisional shells depends on several factors, such as the shear bond strength, physical properties, and costs. According to the results obtained in this study, printing provisional shells and relining them with PMMA yielded an acceptable shear bond strength similar to that when relining self-cure PMMA.

In the present study, the 3D-printed samples underwent a similar post-curing technique, following the manufacturer’s recommendation, in order to standardize the samples; however, it has been shown that post-curing processes can have a significant effect on the mechanical properties and degree of the conversion of the 3D-printed temporary resins. Mayer et al. [27] printed temporary resin samples and cleaned then with different solutions, including 99.5% acetone, pure butyl glycol, 96% ethanol, pure isopropanol, and pure Yellow Magic 7 solution. They measured the degree of conversion, surface roughness, the Martens parameters, and biaxial flexural strength. They observed that the type of cleaning solution had a significant effect on the Martens parameters and biaxial flexural strength of the tested samples. Reymus et al. [28] also evaluated the effect of print layer thickness and post-processing method on the 3D-printed temporary resins. They printed the samples with three different print layer thicknesses of 25 µm, 50 µm, and 100 µm and then placed the samples in four different curing chambers. They measured the degree of the conversion of the printed samples and found that the curing box had a significant effect on the degree of the conversion. Moreover, when compared with that of the 25 µm thickness, they found that the 100 µm and 50 µm printing layer thicknesses had a higher difference between the as-printed degree of conversion and after-cure degree of conversion.

The roughness of the surface has been shown to affect the bond strength of the samples; Kallio et al. [29] found that the grit size of sandpaper has a significant effect on the surface roughness (R_a_) of the tested composite resins. They observed that R_a_ values ranged between 0.41 and 0.91 when the samples were polished with 320 grit size sandpaper, between 0.21 and 0.29 with 800 grit size sandpaper, between 0.08 and 0.20 with 1200 grit size sandpaper, and between 0.03 and 0.05 with 2400 grit size sandpaper. They also found that surface roughness affected the shear bond strength values. In the present study, in order to eliminate the effect of surface roughness on shear bond strength values, all tested samples were polished with the same grit size SiC sandpaper.

The curing of resins on the surface is partially inhibited by the free radical scavengers, including oxygen in the air, generating the so-called oxygen inhibition layer. This layer consists primarily of unreacted monomers and has lower mechanical properties than deeper layers do. Although one might assume that since this layer contains uncured monomers, it would have higher bond strength. Some have shown that the presence of the oxygen inhibition layer does not affect the shear bond strength [30]. In the present study, the oxygen inhibition layer was removed from the surface of all the samples through multiple steps of rinsing, UV curing, and polishing, and it was removed from possible confounding factors interfering with the bond strength results. 

An ideal method to test bond strength between two materials needs to be accurate, reliable, have low technique sensitivity, and be unsophisticated and relatively inexpensive. The most common static bond strength tests are shear, tensile, and push out. Shear bond strength and tensile bond strength tests can be further divided into macro- and micro-tests according to the bonding surface area; macro-tests are defined by a surface area larger than 3 mm^2^, and bond strength micro tests have a bonded surface area of less than 3 mm^2^. Considering the shear bond strength value, micro-tests usually result in higher values when compared with those in shear bond strength macro-tests [31]. Due to the simplicity and lower technique sensitivity, in the present study, a shear bond strength macro-test was used to measure the bond strength value between the printed base and the reline materials [32]. Similarly to the present study, Lankes et al. [20] used a shear bond strength macro-test to test the bond strength to 3D-printed provisional resins. 

One of the limitations of the present study was the relatively small sample size, and further studies with larger sample sizes would be recommended. In addition, in the present study, only two types of 3D-printed resins were tested. Moreover, the present study design did not consider the intraoral environment, including the chewing forces and constant exposure to saliva and oral cavity temperature. These conditions can possibly affect bond strength and need to be addressed in future studies in order to obtain more clinically relevant information on the reline capability of the 3D-printable resins. Hence, future studies with aging mechanisms, including thermocycling or prolonged storage of the specimens, would be highly recommended in order to evaluate the long-term shear bond strength values to 3D-printed temporary resins. It should also be noted that manufacturers are constantly developing new 3D-printable materials and 3D-printing technology is evolving quickly, and possibly at the time of this publication, new resins and/or new technologies may have become available.

## 5. Conclusions

The present study measured and compared the shear bond strength of different reline materials to two available 3D-printing resins approved for long-term provisional restorations. Considering the findings and limitations of this study, the following conclusions may be drawn:

1. Three-dimensional-printed resins for provisional crowns and bridges appear to have a reline strength similar to that of conventional self-cure PMMA provisional material using self-cure PMMA as the reline material.
2. The second highest shear bond strength was obtained by bonding composite resins to the tested 3D-printed resins.
3. Relining the tested 3D-printed resins with bis-acrylic resulted in the lowest shear bond strength value.
4. Both tested 3D-printed resins resulted in similar bond strength regardless of the reline material, suggesting similarities in chemical composition and bonding properties of these resins.
5. Considering the present findings, the need to maintain provisional restorations in the long term in some cases, and the need to modify or characterize the restorations over time, using a self-cure PMMA to reline or add to 3D-printed temporary restorations would be recommended.

## Figures and Tables

**Table 1:** Materials used in this study.

Material	Manufacturer	Type of Material	Application in the Study
E-Dent 400 C&B MFH BL	Envision-Tec	3D-printed resin	Base
NextDent C&B MFH	NextDent	3D-printed resin	Base
Alike	GC	Self-cure polymethylmethacrylate (PMMA)	Base and Reline
Integrity Multicure	Dentsply Caulk	Self-cure Bis-acryl Composite Resin	Reline
Filtek Supreme	3M	Light Cure Composite Resin	Reline

**Table 2:** Shear bond strength values.

Group	N	Shear Bond Strength (MPa)	
		Mean	SD
EP	11	19.54	4.94
EB	11	5.06	1.15
EC	11	14.29	4.19
NP	11	18.80	5.09
NB	11	5.97	2.58
NC	11	12.79	4.32
PP	11	21.89	5.82

**Table 3:** Mode of failure percentages.

Group	N	Adhesive (%)	Cohesive (%)	Mixed (%)
EP	11	3 (27)	0 (0)	8 (73)
EB	11	10 (91)	0 (0)	1 (9)
EC	11	7 (64)	2 (18)	2 (18)
NP	11	1 (9)	2 (18)	8 (73)
NB	11	9 (82)	0 (0)	2 (18)
NC	11	6 (55)	1 (9)	4 (36)
PP	11	1 (9)	0 (0)	10 (91)

## Data Availability

The data presented in this study are available on reasonable request from authors in this article.
